# MADA: a web service for analysing DNA methylation array data

**DOI:** 10.1186/s12859-020-03734-9

**Published:** 2020-11-18

**Authors:** Xinyu Hu, Li Tang, Linconghua Wang, Fang-Xiang Wu, Min Li

**Affiliations:** 1 School of Computer Science and Engineering, Central South University, Changsha, China; 2 Division of Biomedical Engineering and Department of Mechanical Engineering, University of Saskatchewan, Saskatoon, SKS7N5A9 Canada

**Keywords:** DNA methylation, Normalization, Differential methylation analysis, Downstream analysis

## Abstract

**Background:**

DNA methylation in the human genome is acknowledged to be widely associated with biological processes and complex diseases. The Illumina Infinium methylation arrays have been approved as one of the most efficient and universal technologies to investigate the whole genome changes of methylation patterns. As methylation arrays may still be the dominant method for detecting methylation in the anticipated future, it is crucial to develop a reliable workflow to analysis methylation array data.

**Results:**

In this study, we develop a web service MADA for the whole process of methylation arrays data analysis, which includes the steps of a comprehensive differential methylation analysis pipeline: pre-processing (data loading, quality control, data filtering, and normalization), batch effect correction, differential methylation analysis, and downstream analysis. In addition, we provide the visualization of pre-processing, differentially methylated probes or regions, gene ontology, pathway and cluster analysis results. Moreover, a customization function for users to define their own workflow is also provided in MADA.

**Conclusions:**

With the analysis of two case studies, we have shown that MADA can complete the whole procedure of methylation array data analysis. MADA provides a graphical user interface and enables users with no computational skills and limited bioinformatics background to carry on complicated methylation array data analysis. The web server is available at: http://120.24.94.89:8080/MADA

## Background

DNA methylation of cytosine residues at CpG dinucleotides is one of the most extensive studied epigenetic modifications due to its role in biological processes and complex diseases [1]. Developments in high-throughput evaluation of DNA methylation adopting the second-generation sequencing or microarrays technologies have made it possible to quantify DNA methylation of CpG sites in the whole human genome. The prospect of DNA methylation in improving our understanding of disease mechanism such as tumour detection [2] and classification [3] combined with the current trend of expanding coverage and decreasing cost of DNA methylation microarrays has led to the explosive growth of these technologies. Illumina Infinium Human Methylation450 BeadChip (450 k) array is one of the most cost-effective ways to analyse DNA methylation in the human genome, which targets 96% of CpG islands and covers more than 450,000 CpG sites [4]. It has been widely used in many large projects [5], such as The International Cancer Genome Consortium (ICGC) and The Cancer Genome Atlas (TCGA) Project [6]. Recently, a reliable genomic approach Methylation EPIC array to study DNA methylation levels and patterns in the whole human genome has developed by Illumina, which covers more than 850,000 CpG sites. As methylation arrays may still maintain its popularity for detecting methylation in the predictable future, so it is significant to provide comprehensive workflow for DNA methylation array analysis.

In recent years, a lot of tools have been proposed to analyse methylation array data with the availability of public data resources. However, most of the tools provided to users are based on command line tools published on Bioconductor. The most popular command line-based platform is Minfi [7], which provides a flexible analysis for Infinium DNA methylation microarrays. However, the complex usage of Minfi causes a significant challenge to the researchers without proficient programming skills. The Bioconductor package ChAMP [8] also has the similar problems. Many biological web platforms [9, 10] provided convenient input and output for biologists. Only a few tools are based on methylation array data, such as ADMIRE [11] integrates several normalization methods and provides a statistical method for differentially methylated regions (DMRs), which can only handle 450 k data.. Furthermore, most of these web-based tools can neither cover the whole process of methylation array data analysis, nor provide downstream analyses and result visualization.

Here, we develop a web-based tool MADA which provides a comprehensive analysis of methylation arrays data by integrating quality control, filtering, nine normalization methods, five differential methylation analysis methods and two downstream analysis methods. MADA has good usability and practicability can not only handle the classic methylation array data of 450 k, but also analyse the emerging EPIC data. MADA places great emphasis on workflow, which helps finishing the whole analysis through several easy steps. In addition, MADA provides visualization for the entire process, including the results of pre-processing, differentially methylated probes (DMPs) or regions, gene ontology and pathway analysis.

## Implementation

### User-friendly graphical user interface

**Fig. 1:**
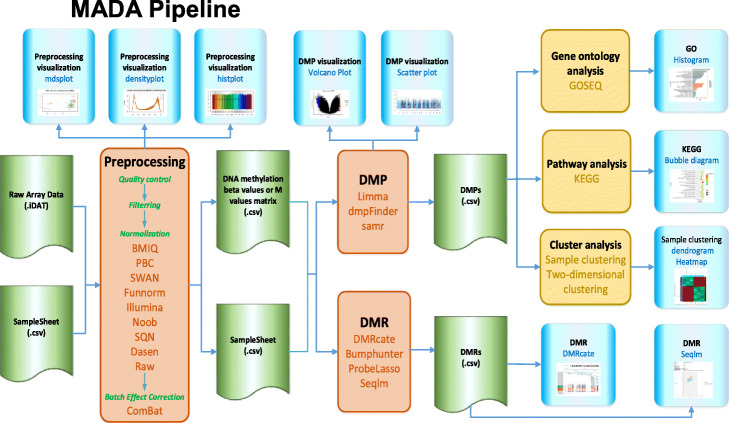
MADA Pipeline. It includes four stages: Pre-processing (Quality controls, Filtering, Normalization, batch effect correction), DMPs, DMRs and downstream analysis. The visualization of Pre-processing, DMP, DMR, and downstream analysis are also provided

MADA provides a graphical user interface and enables users with no computational skills and limited bioinformatics background to carry on the analysis of sophisticated methylation array data. Figure 1 shows the pipeline of MADA. The web server allows users to define workflow on their own, and to complete their analysis through several easy steps. First, users upload custom methylation array datasets including the raw iDAT files and a sample sheet file which describes important sample information in detail. Second, users can adjust a wide range of analysis parameters, such as quality control filtering based on detection *p* values, the aim of filtering is to remove probes on the sex chromosome, remove probes with SNPs or exclude cross reactive probes, nine normalization methods, batch effect correction, three DMPs detecting methods, four DMRs detecting methods, and visual diagram is provided for each step. Furthermore, pre-processed methylation beta values or M values matrix files can be uploaded directly to detect DMPs or DMRs. If users are interested in the downstream analysis of DMPs, GO analysis, pathway analysis and cluster analysis can be used to explore biological significance. After submitting the task, users will receive a notification about the job status and a URL of analysis result, on which all analysis results are generated in the web server and can be downloaded as a compressed archive from the MADA platform.

### Data pre-processing and batch effect correction

The step of data pre-processing is necessary in the overall data analysis process. First, MADA has implemented quality control for raw data which calculates detection *p*-values for each CpG in each sample using the Minfi package [7]. Low quality samples and probes can be easily removed from the analysis by setting the detection p-value in advance. Second, MADA integrates nine typical normalization methods, including BMIQ [12], PBC [13], SWAN [14], Funnorm [15], Illumina [7], Noob [16], SQN [17], Dasen [18], and Raw to decrease the technical variation within and between arrays. Beta Mixture Quantile normalization (BMIQ) is a flexible approach by quantile normalization to fit the distribution of beta-values of Infinium II to the corresponding distribution of beta-values of Infinium I, which seems more suitable to be used to normalize data than other methods. Peak-based correction (PBC) method is sensitive to the shape of beta-value density curves which rescales the methylation values of Infinium II to the same modes for methylation values distribution of Infinium I. Subset-quantile Within Array Normalization (SWAN) is a within-array normalization method by altering Infinium I probe data to increases the technical variation. Functional normalization (Funnorm) extends the quantile normalization method to removes unwanted technical variation using control probes. Illumina is not only a within-array normalization but also a between-array normalization method performed pre-processing as Genome Studio. Noob is a novel approach to background correction for Infinium Human Methylation data to account for technical variation in background fluorescence signal. Subset Quantile Normalization (SQN) assumes that the beta values of CpGs from the same biological category should obey the same density distribution. Dasen is a data driven analysis method to preprocess 450 K methylation array data. Table S1 of Supplementary Materials 1 lists the normalization tools and corresponding performance. Moreover, MADA provides the function of filtering to exclude probes on the sex chromosomes and probes with SNPs [7] at CpG site, and exclude cross reactive probes [19] in the step of pre-processing.

Due to some potential biological and environmental variables that cannot be measured and may have a significant effect on the measurements of high-throughput biological experiments. MADA implements the batch effect correction with ComBat method from the sva package [20] which was used to further remove technical bias introduced by interrogating samples on the methylation array in different batches. The ComBat function uses an empirical Bayesian framework to adjust to known batches. Eliminating batch effects in differential analysis have been shown highly effective in reducing dependence and stabilizing error rate estimates.

### Differential methylation analysis

After pre-processing, the main purpose of many methylation researches is detecting differentially methylated probe (DMPs) or differentially methylated regions (DMRs) in the human genome. Differential methylation analysis is the most important step, which takes the output file of pre-processed results by different methods as input. Seven popular differential methylation analysis methods (Limma [21],dmrFinder [7], samr [22], DMRcate [23], Bumphunter [24], ProbeLasso [25], seqlm [26]) are integrated in MADA, which based on different statistical methods as shown in Table S2 of Supplementary Material 1. Three of them are used to detect DMPs, Limma is a R package for analysing gene expression and methylation data from microarray technologies and the linear model is considered as the most popular and widely accepted tools for analysing designed experiments and the assessment of differential expression. DmpFinder is a function of R package Minfi which tests each genomic position for the association between methylation and phenotype. Samr is a package for analysing microarrays data, which correlates many features with an outcome variable, such as a quantitative variable, group indicator or survival time. The remaining four were used to detect DMRs. DMRcate is a novel tool based on a combination of DM-signal smoothing and subsequent threshold specification to agglomerate genomically localized individual DNA methylation CpG sites into discrete DMRs. Bumphunter is a flexible approach for identifying DMRs of biological interest based on quantitative high-throughput methods. ProbeLasso is a novel approach to rope in DMRs with DNA methylation array data. Seqlm is an MDL based method for DMR identification by using linear mixed models.

### Downstream analysis and visualization

Go analysis, pathway analysis and cluster analysis are implemented in MADA, which help users to conduct further analysis and discover significant results behind these data. In order to highlight biological processes and reduce complexity, Go analysis is widely used in genome-wide expression studies. GOseq [27] is a software that includes functions to calculate the significance for each Go category amongst differentially expressed genes. MissMethyl [28] provides a KEGG pathway analysis using the gometh function. R basic clustering function is used in cluster analysis. Downstream analysis uses the output result of DMPs as input, which contains a long list of important CpG sites. Moreover, MADA provides the visualization of pre-processing, DMPs or DMRs, gene ontology and pathway analysis results making use of R, and all visual images can be downloaded on a web page. These tools are listed in Table S3 of Supplementary Material 1.

## Results

### Case study of 450 K data: DNA methylation changes in endometrium and correlation with gene expression during the transition from pre-receptive to receptive phase

**Fig. 2:**
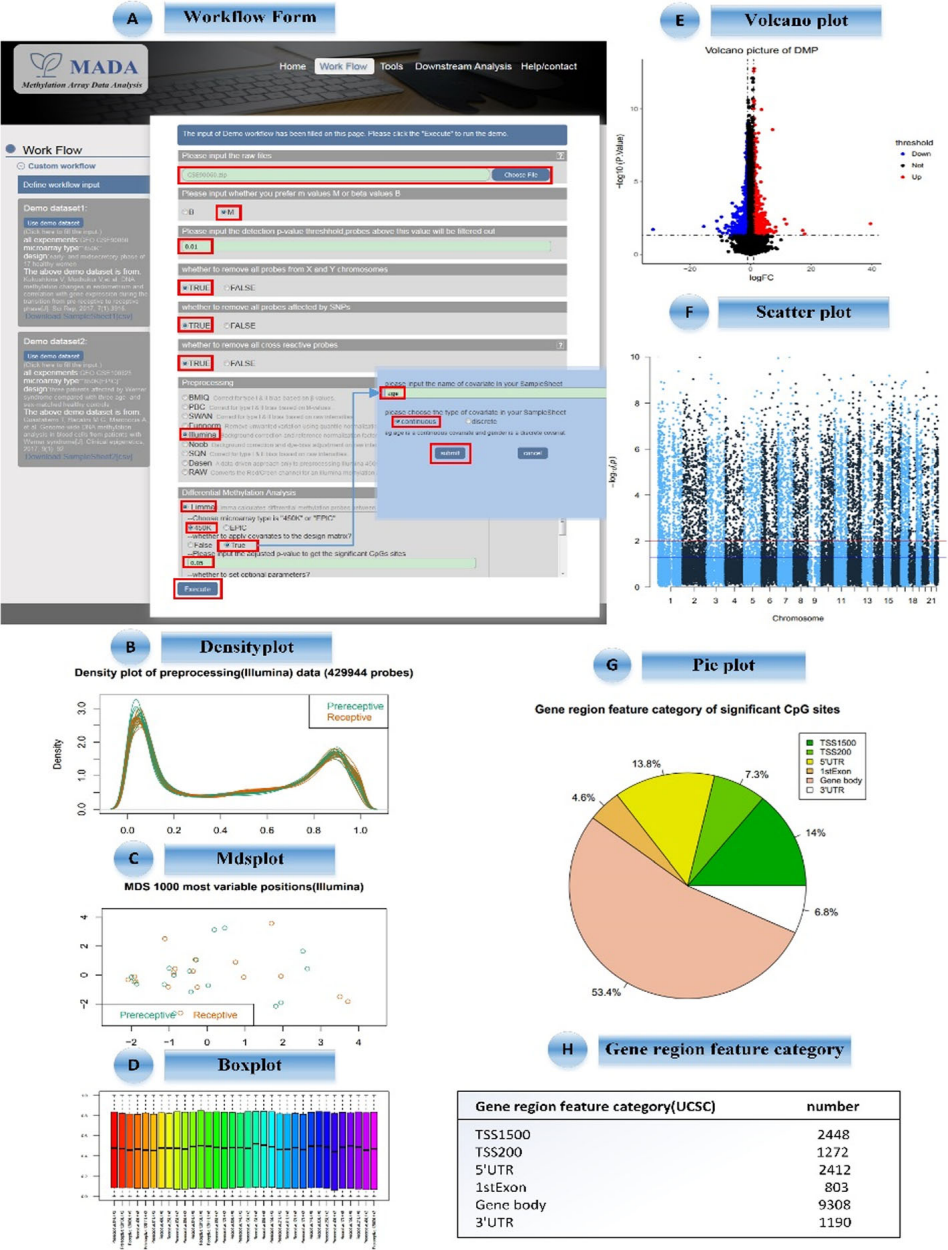
MADA web application interface and case results. **a** WorkFlow form, where the user uploads their custom datasets and submits the request of processing. **b** Densityplot shows DNA methylation levels (as β values) for pre-receptive (LH + 2) and receptive (LH+ 8) endometrium samples from 17 women. **c** Mdsplot drawn with the largest difference between the first 1000 samples, can be used to reflect the similarity of samples. **d** Boxplot shows the data of each chip is still tidy so that we can detect DMPs or DMRs in the next step. **e** Volcano plot shows the degree of difference in CpG sites within different methylation periods. **f** Scatter plot can be used to reflect the distribution of CpG sites on chromosomes. **g** Pie plot shows the gene region feature category (UCSC) of significant CpG sites, and the percentage of significant CpG sites in different gene annotation region directly. **h** More detailed numerical information can be seen in table

This case study describes the approach reported in Scientific Reports [29]. We repeated the parts of pre-processing and differential methylation analysis in their Methylation array data analysis. In this study, the relationship between DNA methylation and gene expression in endometrial biopsies of 17 normal women of childbearing age was studied by genome-wide technique. Seventeen healthy women of childbearing age received pre-receptive and receptive periods in one menstrual cycle. Here we used the “WorkFlow” module to repeat this analysis process. Firstly, Methylation dataset was downloaded from Gene Expression Omnibus (GEO) [30] under accession number of GSE90060. We can get the raw iDAT data and a samplesheet.csv (see ‘Help/Contact’ in website). On the page of “WorkFlow”, step by step we selected “M-value” to conduct differential methylation analyses, and input the detection *p*-value 0.01 as a threshold to filter out unreliable probes, and then chose “TRUE” to remove all probes from X and Y chromosomes affected by SNPs. The pre-processing box contains 9 normalization tools integrated in MADA, here we chose Illumina with the default parameters. The differential methylation analysis box contains 7 differential detection tools, we chose Limma with the default setting, and chose the continuous covariates of age for the design matrix, microarray type is “450 K”. The last step, after clicking the “Execute” button, the workflow starts. The definition of the workflow is shown in Fig. 2a. When the operation is finished, the web jumps to the page of “RecentJob” and users can download the final results to their local computer in zip file. In order to compare these results more obviously, we set the adjusted *p*-value as 0.05 to get the same 21,117 significant CpG sites in paper results [29]. The significant CpG sites associated with over 5000 differentially expressed genes between pre-receptive and receptive endometrium as show in Supplementary Material 2.

The visualization of pre-processing is as shown in Fig. 2c-d. Densityplot shows DNA methylation levels (as β values) for pre-receptive (LH + 2) and receptive (LH+ 8) endometrium samples from 17 women. Mdsplot drawn with the largest difference between the first 1000 samples, which can be used to reflect the similarity of samples. From boxplot we can see, the data of each chip is still tidy so that we can detect DMPs or DMRs in the next step. The visualization of DMP is shown in Fig. 2e-g. Volcano plot shows the degree of difference in CpG sites within different methylation periods. Scatter plot can be used to reflect the distribution of CpG sites on chromosomes. From pie plot we can see the gene region feature category (UCSC) of significant CpG sites, and the percentage of significant CpG sites in different gene annotation region directly. More detailed numerical information can be seen in Fig. 2h. Sometimes a CpG site can correspond to multiple genes so that may repeatedly correspond to multiple gene regions. All significant CpG sites information are shown in Supplementary Material 2.

### Case study of EPIC data: genome-wide DNA methylation analysis in blood cells from patients with Werner syndrome

The second case form Clinical Epigenetics [31] uses the Infinium Human Methylation 850 K BeadChip arrays to verify whether epigenetic changes are associated with Werner syndrome phenotype. As the operation of the workflow mentioned above, step by step we selected beta value to conduct differential methylation analyses, and input the detection *p*-value 0.01 as a threshold to filter out unreliable probes, and chose “TRUE” to remove all probes from X and Y chromosomes. Then we chose Funnorm function with the default parameters in pre-processing box. In differential methylation analysis box, we chose Limma with the default setting, chose “False” to apply covariates to the design matrix and chose microarray type as “EPIC”. When the operation is finished, the web jumps to the page of “RecentJob” and users can download the final results to their local computer in a zip file, which contains files as same as case study 1. To verify the effectiveness and reliability of the platform, we list the same results of the top 20 DMPs as the case from Guastafierro’s paper which is shown in Table 1.

**Table 1:** List of the top 20 DMPs

Probe	chr	pos	Relation_to_Island	UCSC_RefGene_Name	Islands_Name
cg15294279	3	174,842,010	OpenSea	NAALADL2	
cg00597723	5	158,691,793	S_Shore	UBLCP1	chr5:158690013–158,690,541
cg16995742	2	237,992,612	N_Shore	COPS8;	chr2:237994004–237,994,876
cg13956086	7	2,434,521	OpenSea		
cg23432430	12	125,538,377	S_Shelf		chr12:125534060–125,534,527
cg26845082	3	13,555,664	OpenSea		
cg17779733	22	49,589,242	OpenSea		
cg23928292	12	21,815,474	OpenSea		
cg06052372	16	83,967,808	OpenSea		
cg10360725	8	1.44E+ 08	OpenSea		
cg13885829	1	17,482,041	OpenSea		
cg14782559	6	33,131,893	S_Shelf	COL11A2;	chr6:33129291–33,129,718
cg26822175	22	27,018,010	OpenSea	CRYBA4	
cg20757478	6	31,012,262	OpenSea		
cg18673341	7	22,481,962	OpenSea	MGC87042;	
cg17628377	5	180,121,337	OpenSea		
cg08161337	22	45,814,116	OpenSea	RIBC2	
cg15865722	11	68,860,657	OpenSea		
cg07584620	1	2,265,881	N_Shore	MORN1	chr1:2266007–2,266,432
cg22664298	5	128,795,827	Island	ADAMTS19	chr5:128795503–128,797,417

### Comparison of MADA with other typical platforms

Table 2 shows a comprehensive comparison between MADA and several other typical platforms for Methylation array data analysis including Minfi, ChAMP, watermelon, RnBeads, ADMIRE, Methylumi, missMethyl. Table 2 also lists the systems that the platforms rely on, the installation requirement, the interface, and the functions. As shown in Table 2, MADA has a user-friendly interface, and provides visualization for the entire process, compared with command line-based platforms. In addition, MADA has no requirement for the operating environment, compared with GUI-based platforms. Moreover, MADA integrates more complete methylation array data analysis procedures, and allows users to define their own workflow, compared with other web-server based platforms.

**Table 2:** Current typical platforms for methylation array data analysis

platform	system	installation	Interface		Functions & tools						
				workflow	Preprocessing	DMP	DMR	Data visualization	Gene ontology analysis	Pathway analysis	Cluster analysis
**Minfi** [7]	Unix/Linux, Mac OS, Windows	R package manager	Command line	**–**	Funnorm, SWAN, Illumina, SQN	**–**	Bumphunter	Preprocessing	**–**	**–**	**–**
**ChAMP** [8]	Unix/Linux, Mac OS, Windows	R package manager	Command line interface, GUI	**√**	BMIQ.PBC SWAN, Funnorm,Combat	Limma	DMRcate, Bumphunter, ProbeLasso	Preprocessing, DMP/DMR/GO	**√**	**√**	**–**
**watermelon** [18]	Unix/Linux, Mac OS, Windows	R package manager	Command line	**–**	Dasen, BMIQ, SWAN	**–**	**–**	**–**	**–**	**–**	**–**
**RnBeads** [32]	Unix/Linux, Mac OS, Windows	R package manager	command line	**√**	SWAN, Noob, Dasen	Limma		Preprocessing	**√**	**–**	**–**
**ADMIRE** [11]	Unix/Linux, Mac OS, Windows	not needed	Web server/Command line	**–**	Funnorm, SWAN, Illumina, SQN	**–**	A statistical testing	Preprocessing, DMR	**√**	**–**	**–**
**Methylumi** [16]	Unix/Linux, Mac OS, Windows	R package manager	command line	**–**	Noob	**–**	**–**	Preprocessing	**–**	**–**	**–**
**missMethyl** [28]	Unix/Linux, Mac OS, Windows	R package manager	Command line interface	**–**	SWAN	Limma	**–**	Preprocessing DMP	**√**	**√**	**–**
**MADA**	Unix/Linux, Mac OS, Windows	not needed/compilation from script	Web server/local web server	**√**	BMIQ, PBC, Noob, Funnorm, SWAN, Illumina, SQN, RAW, Combat	Limma	DMRcate, Bumphunter, ProbeLasso, Seqlm	Preprocessing DMP/DMRGO/KEGG	**√**	**√**	**√**

## Conclusion

With the widely use of methylation array data in exploring the associations between DNA methylation and complex diseases, more efficient tools are in urgent need to process HM450K and EPIC array data. In this study we propose a comprehensive pipeline for Illumina methylation array data analysis. The major contributions of the present work lie in MADA provides more comprehensive functions, from pre-processing to differential methylation analysis, and the tools used to downstream analysis are also integrated. MADA further provides the visualization of pre-processing, differentially methylated probes or regions, gene ontology, and pathway analysis results. Besides, MADA allows users to define workflow on their own, and to complete their analysis through several easy steps. MADA encourages researchers to design their customized experiment such as trying different pre-processing methods or combining them to get the best results and use existing datasets to reveal vital new information.

With the analysis of two examples, we have shown that MADA has good usability and practicability can not only handle the classic methylation array data of 450 K, but also analyse the emerging EPIC data. The first case can help researchers to understand the molecular mechanisms governing endometrial biology and receptivity, which highlights the need for similar studies in distinct endometrial cell populations. The second shows DNA methylation changes in the peripheral blood from Werner syndrome patients, which can provide a new insight in the pathogenesis of the disease and help researchers to understand a functional correlation of gene expression and methylation status in some cases.

MADA is meant for the whole process of methylation array data analysis. Whereas most of existing analysis tools are based on command-line published on Bioconductor. MADA provides a user-friendly web-based service as well as source code. However, public web services typically carry out very limited operations on throughput because the workload has to be regulated by the website provider. To solve the above difficulties, MADA will continue to explore advanced strategies and innovative approaches. MADA can potentially provide more smart services such as RNA-seq based conjoint analysis, methylation data based classification of schizophrenia, etc.

## Supplementary information

**Additional file 1: Supplementary Material 1.** This file contains three tables of the list of pre-processing tools, differential methylation analysis tools, differential methylation analysis tools integrated in MADA.

**Additional file 2: Supplementary Material 2.** This file contains the same significant differentially methylated CpGs from the MADA and the paper of Kukushkina et al.

## Data Availability

The datasets used in case study are available in accession GSE90060 and GSE100825. MADA is an open source collaborative initiative available in the GitHub repository: https://github.com/huxinyu/Methylation. The supplementary materials are provided, and the web server is available at: http://bioinformatics.csu.edu.cn/MADA/.Just in case, users can also access MADA at: http://120.24.94.89:8080/MADA.

## Abbreviations

MADA: Methylation arrays data analysis
DMPs: Differentially methylated probes
DMRs: Differentially methylated regions
450 K: Illumina Infinium Human Methylation450 BeadChip array
EPIC: Illumina Infinium Human Methylation EPIC array
