# Supplementary material for: MADA: a web service for analysing DNA methylation array data
Source: BMC Bioinformatics. 2020 Nov 18;21(Suppl 6):403. doi: 10.1186/s12859-020-03734-9 (PMC7672854; doi:10.1186/s12859-020-03734-9)
Supplement: Supplementary file 1 — Additional file 1: Supplementary Material 1. This file contains three tables of the list of pre-processing tools, differential methylation analysis tools, differential methylation analysis tools integrated in MADA. [file 12859_2020_3734_MOESM1_ESM.doc]

Supplementary Material

Table S1 The list of pre-processing tools integrated in MADA

| **Tool** | **Performance** |
| --- | --- |
| **BMIQ** | BMIQ is a mixture-model-based normalization method designed to correct the type II probe bias and make the methylation distribution of type II features comparable to the distribution of type I features. |
| **PBC** | PBC is a peak-based correction method to estimate the M-value peaks for Infinium I and II independently, and rescale the Infinium II values to match the Infinium I initial range. PBC is also a within-array normalization method. |
| **SWAN** | SWAN is a within-array normalization method based on the assumption that probes with the same number of CpGs in the probe body should have similar intensity distributions (since the regions they interrogate should have similar biological features) |
| **Illumina** | Illumina is not only a within-array normalization but also a between-array normalization method performed preprocessing as Genome Studio. Background correction and reference normalization factor based color-channel normalization. |
| **Noob** | Noob performs within-array normalization to correct for background fluorescence and dye bias. It fits a normal-exponential convolution model to estimate the true signal conditional on the observed intensities. |
| **SQN** | SQN is another between-sample normalization method for methylated and unmethylated intensities that also involves within-sample normalization of type I and II probes. |
| **Dasen** | Dasen is a between-sample normalization method, like Noob and SWAN, it adjusts the raw intensities instead of Beta values |
| **Funnorm** | Funnorm is a between-sample (functional) normalization method that attempts to remove unwanted variation by adjusting for covariates estimated from a control probe matrix. |
| **Raw** | Raw converts the Red/Green channel for an Illumina methylation array into methylation signal, without using any normalization |

Table S2 The list of differential methylation analysis tools integrated in MADA

| **Tool** | **Performance** |
| --- | --- |
| **Limma** | Limma provides an integrated solution for analyzing data from gene expression experiments. limma contains rich features for handling complex experimental designs and for information borrowing to overcome the problem of small sample sizes. It also contains particularly strong facilities for reading, normalizing and exploring such data |
| **DMRcate** | DMRcate fits replicated methylation measurements from the Illumina HM450K BeadChip (or 450K array) spatially across the genome using a Gaussian kernel. DMRcate identifies and ranks the most differentially methylated regions across the genome based on tunable kernel smoothing of the differential methylation (DM) signal. |
| **Bumphunter** | Bumphunter allows accomplish bump hunting in genomic data. bumphunter addresses batch effects, exploits the correlation structure of the microarray data to identify differentially methylated regions (DMRs), and provides a genome-wide measure of uncertainty. It was applied to microarray data and was able to identify epigenomic regions of biological interest. |
| **ProbeLasso** | ProbeLasso is a flexible window based approach that gathers neighboring significant-signals to define clear DMR boundaries for subsequent in-depth analysis. |
| **Seqlm** | Seqlm is a method for identifying differentially methylated regions. First, the data is divided into smaller segments based on genomic distance between consecutive probes. Then, each of these segments is divided into regions with consistent differential methylation patterns. For this, all possible segmentations are considered and the optimal one is chosen according to the minimum description length (MDL) principle. |

Table S3 The list of downstream analysis (GO/KEGG) tools integrated in MADA

| **Tool** | **Performance** |
| --- | --- |
| **GOseq** | GOseq allows users to perform gene ontology (GO) analysis on array data. GOseq is a software system that includes functions for calculating the significance of over-representation of each GO category amongst DE genes. These functions give researchers the possibility to select which type of bias they wish to compensate for, between two options: transcript length bias or total read count bias. |
| [**missMethyl**](http://www.bioconductor.org/packages/release/bioc/html/missMethyl.html) | MissMethyl allows users to perform KEGG pathways analysis. In addition, [missMethyl](http://www.bioconductor.org/packages/release/bioc/html/missMethyl.html) performs normalization, removal of unwanted variation in differential methylation analysis, differential variability testing and gene set analysis for the 450K array. |
| **hclust** | This function performs a hierarchical cluster analysis using a set of dissimilarities for the n objects being clustered. |
